# Medicaid Home and Community Based Services Spending and Nursing Home Use by Individuals Under the Age of 44

**DOI:** 10.1177/00469580251323779

**Published:** 2025-03-12

**Authors:** Seiyoun Kim, Ziwei Pan, Nurah Koney-Laryea, Hye-Young Jung, Sophia Jan, Kira L. Ryskina

**Affiliations:** 1 Leonard Davis Institute of Health Economics, Philadelphia, PA, USA; 2 Morehouse School of Medicine, Atlanta, GA, USA; 3 Weill Cornell Medical College, New York, New York, USA; 4 Northwell Health, New Hyde Park, NY, USA; 5 Cohen Children’s Medical Center, New Hyde Park, NY, USA; 6 Perelman School of Medicine at the University of Pennsylvania, Philadelphia, PA, USA

**Keywords:** HCBS, nursing home, young adults, children, racial disparities

## Abstract

Prior studies of the role of state spending on home and community-based services (HCBS) in nursing home use focused on adults over the age of 65. However, medically complex children and adults under 50 years old represent a small (about 5%) but highly vulnerable subset of nursing home patients. We measured the impact of HCBS spending on short-term and long-term nursing home stays by children and adults under 44 years old and compared the impact between Non-Hispanic White (NHW) individuals and Black, Indigenous, and People of Color (BIPOC). We used the Minimum Data Set to measure nursing home stays in each state per year in 2012 to 2019. The Medicaid Long Term Services and Supports annual expenditure reports were used to measure HCBS expenditures per state resident with a disability. Our outcome was nursing home use by children (<18 years old) and adults (18-43 years old) associated with a change in HCBS expenditures per state resident with a disability (measured in $1000 increments) estimated using linear regression. Higher HCBS expenditures per resident were associated with fewer short-term and long-term nursing home stays among NHW children. We did not find statistically significant association between changes in HCBS expenditures and nursing home stays among BIPOC children. Investments in HCBS are necessary to reduce nursing home use among younger adults. However, to mitigate racial disparities in nursing home use among children, HCBS spending alone may not be sufficient.

## Introduction

Home and Community Based Services (HCBS) make up more than half of Medicaid long-term care expenditures, providing critical support to people with chronic debilitating conditions.^1^ Comprised of home health care, personal support services, adult day care, nursing, nutritional services, and durable medical equipment, these resources help individuals avoid institutionalization or facility-based care, which is typically less personalized to an individual’s needs and more expensive. By offering care in a familiar setting, HCBS promote patient autonomy, support long-standing social connections in the community, and facilitate more personalized care tailored to individual needs. In fact, prior studies found that higher per-beneficiary HCBS spending in a state is associated with more adults with severe functional impairment residing in the community as opposed to a long-term care nursing home,^2,3^ and lower nursing home use among adults who are 65 or older without children.^4^ However, prior studies of the impacts of HCBS spending on nursing home use focus on adults over the age of 65.

Less is known about the role of HCBS spending in nursing home use among children and younger adults. Medically complex children and adults under 50 years old represent approximately 3% of long-term nursing home residents,^5^ and 1 in 10 short-stay admissions. Younger individuals with complex care needs are clinically and socially distinct from the older adults who represent the majority of nursing home patients.^5^ A 2022 study comparing characteristics of nursing home use for individuals under 65 years of age to those over 65 years old reported differences in demographics, clinical characteristics, and the characteristics of nursing homes where they receive care. Moreover, most adults over 65 receive coverage for short-term nursing home use and other services through Medicare, which does not cover long-term stays or long-term services and supports in the community. Hence, the findings of prior studies focusing on older populations may not reflect the relationship between HCBS spending and nursing home use for children and younger adults.

Furthermore, the effects of HCBS spending may vary by race and exacerbate existing racial and ethnic disparities in nursing home use among children and younger adults. HCBS spending does not address the racial wealth gap that contributes to disparities in access to caregiver resources between Black and White families.^6^ Moreover, studies of family and unpaid caregivers reported differences in the caregiving experience between Black and White caregivers,^7,8^ highlighting the need for more equitable caregiver support policies. Prior studies of Black, Indigenous, and People of Color (BIPOC) parents of clinically complex children report poor access to advocacy groups, consumer directed personal care services, and language barriers, which may lead to increased pressure to use facility-based care for their children.^9-11^ Prior studies report differences in the impact of HCBS spending between White and non-White older adults. Counties with higher spending on HCBS between 2010 and 2013 experienced fewer nursing home stays among patients with dementia who were White but increased nursing home use among patients with dementia who were Black.^3,12^ The impacts of HCBS spending on racial disparities in nursing home use among younger individuals are less well understood.

Our objectives in this paper are 2-fold. The first aim is to measure the impact of HCBS expenditures on nursing home use among the following 2 groups: children (<18 years old), and younger adults (18-43 years old). Children under 18 years old face a distinct landscape of resources for skilled nursing and custodial care in the community compared to the resources available to adults between the ages of 18 to 43 years old. For instance, children receive federally mandated school-based services and the Early and Periodic Screening, Diagnostic and Treatment (EPSDT) benefit.^13^ As these individuals enter young adulthood, they face increased exposure to variations in state-level HCBS spending. Thus, we hypothesize that HCBS expenditures will have less impact on children less than 18 years old compared to adults between 18 and 43 years old. The second aim of this paper is to compare the impacts of HBCS spending on nursing home use by children and adults under 44 years of age between 2 groups: BIPOC and Non-Hispanic White (NHW) individuals. We hypothesize that changes in HCBS expenditures will have less impact on BIPOC compared to NHW individuals, exacerbating the racial disparities in nursing home use. Our hypothesis is based on evidence from prior studies among older adults,^3,12^ and because HCBS expenditures represent a blunt instrument that does not address the many causes of racial disparities in caregiving for medically complex younger individuals such as access to advocacy groups, personal care services, nor mitigate language barriers.

## Methods

### Data and Sample

We use the nursing home Minimum Data Set (MDS) from 2012 to 2019 to measure short and long-term nursing home use by children and adults under 44 years of age. We excluded hospital-based nursing homes from the study sample because the characteristics of the residents in these facilities differ greatly from those in freestanding nursing homes. The total number of short-term nursing home stays over our study period was 2 773 330 and the total number of long-term nursing home stays was 1 623 907. Demographic information such as age, race, and gender are also collected from the MDS. We use the Medicaid Long Term Services and Supports (LTSS) annual expenditure reports^1,14-20^ to obtain HCBS expenditures by year and state. State level demographic characteristics including the total number of residents with disabilities,^21^ median household income,^22^ and percentage of population below the poverty level^23^ are obtained from the US Census. State level nursing home characteristics such as total number of beds, profit status and occupancy rate are obtained from Brown University’s LTCFocus database.^24^

### Variables

Our main outcome of interest is the number of nursing home stays by children less than 18 years old and adults 18 through 43 years old in each state and year. We count short-term and long-term care stays separately, because those services have different therapeutic goals and may be differentially affected by HCBS spending. We also measure the number of stays separately for Non-Hispanic White (NHW) and Black, Indigenous, and People of Color (BIPOC) individuals. Thus, we count the number of nursing home stays in the following 4 subgroups: (1) NHW children less than 18 years old, (2) NHW adults between 18 and 43 years old, (3) BIPOC children less than 18 years old, and (4) BIPOC adults between 18 and 43 years old.

Although the MDS includes 6 race and ethnicity categories: American Indian or Alaskan Native, Asian, African American, Hispanic, Native Hawaiian or Pacific Islander, and White, the narrower racial and ethnic categories result in sample sizes of fewer than 10 observations in some years and age groups. Therefore, we combine the American Indian or Alaskan Native, Asian, African American, Hispanic, Native Hawaiian or Pacific Islander subgroups in the BIPOC category.

Federal regulations require nursing homes to conduct patient assessments at regular intervals, but the discharge assessments are missing for many short-term stay patients. For this reason, we use the following approach to distinguish between short-term stays and long-term stays. Individuals with a quarterly assessment within 100 days of the admission assessment are considered long-term care patients, consistent with prior work.^25^ All others are considered short-stay nursing home patients.^25^

We measure HCBS spending per state resident with a disability by dividing the total expenditures by the number of state residents with a disability in each state. The total number of residents with disabilities was collected from the American Community Survey which considers disability as any hearing, vision, cognitive, ambulatory, self-care, or independent living difficulty.^21^ We then adjust the spending for inflation using the adjustment factor for medical care services. Spending is reported in 2012 dollars.^26^

### Analysis

We first describe state level characteristics based on the quartiles of HCBS spending per individual with a disability. The characteristics included were percentage of for-profit nursing homes within the state, average nursing home occupancy rate, total number of beds across all nursing homes in the state, median household income, and the percentage of individuals living below the poverty level. We then summarize nursing home use by age (less than 18 years old, between 18 to 35 years old, and between 36 and 43 years old), and for the NHW and BIPOC subgroups, by quartile of HCBS spending.

We use linear regression with state and year fixed effects to estimate the association between a change in HCBS spending and the number of nursing home stays. In the first model we do not adjust for covariates. Because other variables known to affect nursing home use may confound the relationship between HCBS spending and our outcomes, we estimate a second set of models, adjusted for the following additional variables that might impact nursing home use, HCBS spending or both: the total number of residents with a disability, the total number of nursing home beds, percentage of for-profit nursing homes, nursing home occupancy rate, median income, and percentage of population below the poverty line. We include the percentage of for-profit nursing homes in a state, because for-profit facilities operate under different financial incentives compared to non-profit facilities and tend to devote fewer resources to direct patient care,^27^ which potentially can impact nursing home use. The standard errors are clustered at the state level.

To address concerns about inadequate reporting of Medicaid HCBS spending, particularly with the rise of managed care, we performed sensitivity analyses excluding states previously identified to have a high degree of missingness in the expenditure data.^1,19^ Specifically, we excluded California and New Mexico for 2012; New Mexico and North Carolina for 2013; California, Massachusetts, and North Carolina for 2014; California, North Carolina, and Michigan for 2015; California, South Carolina, and Michigan for 2016; and California, Delaware, Illinois, and Virginia for 2017 through 2019.

All analysis was performed using Stata MP, version 18 (StataCorp LLC). The study is exempt from review by the University of Pennsylvania IRB because the data are aggregated at the state level and do not meet the definition of human subjects research. This study follows the Strengthening the Reporting of Observational Studies in Epidemiology (STROBE) reporting guidelines.

## Results

**Figure 1.**
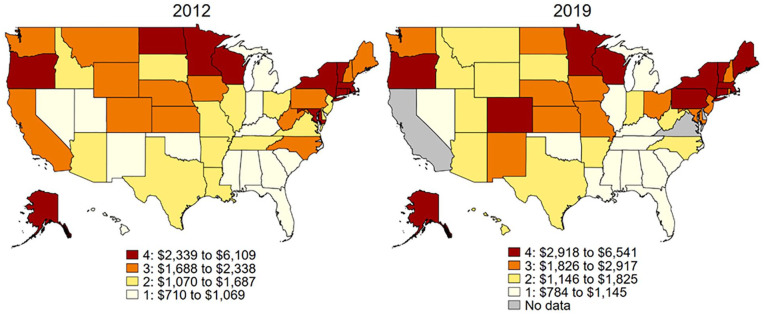
Heat map of HCBS expenditures per state resident with disability by quartile, 2012 and 2019. *Note.* States are categorized by quartile of HCBS expenditures per state resident with disability. Darker colors indicate higher spending.

**Figure A1.**
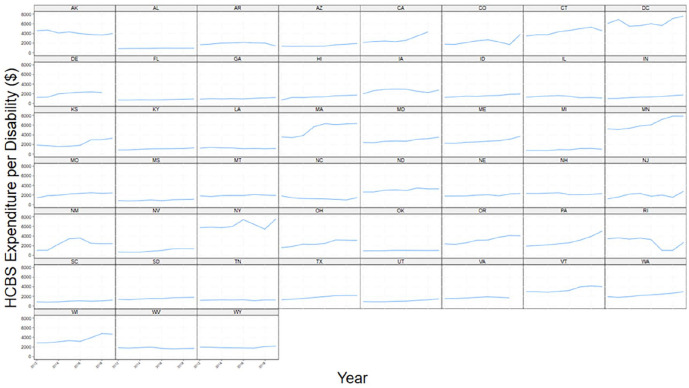
HCBS expenditures per state resident with disability by state: 2012 to 2019.

Between 2012 and 2019, HCBS spending trends differed between states. To provide an overview of the differences in spending between states and over time, Figure 1 displays heat maps corresponding to each quartile of HCBS spending for years 2012 and 2019. Appendix Figure A1 shows the exposure variable—the change in inflation adjusted HCBS spending per individual with a disability for each state from 2012 through 2019. In general, 16 states increased HCBS spending over the time period. One state reduced its HCBS spending, and the rest of the states' spending either remained stable or fluctuated over time (Appendix Figure A1).

Table 1 shows the state level characteristics categorized by quartiles of HCBS spending per individuals with a disability for years 2012 and 2019. The mean spending by quartile ranged from $867 for the bottom quartile to $3787 for the top quartile in 2012. Spending increased across all quartiles. In 2019, the mean HCBS spending was $950 for the bottom quartile and $4394 for the top quartile. States in higher quartiles of HCBS spending had a higher median household income and a lower percentage of state residents living below the poverty level (Table 1).

**Table 1.** State Characteristics by Quartile of HCBS Expenditures per State Resident With Disability.

State characteristics	2012				2019			
	Quartile 1	Quartile 2	Quartile 3	Quartile 4	Quartile 1	Quartile 2	Quartile 3	Quartile 4
HCBS expenditure per disability, mean $ (SD)	867 (118)	1367 (162)	1963 (181)	3787 (1308)	950 (112)	1499 (214)	2357 (346)	4394 (1342)
Residents with disability, mean N (SD)	773 735.7 (616 596.5)	903 515.6 (758 866.3)	757 485.2 (1 037 357.8)	498 453.8 (566 175.8)	1 046 017.6 (677 758.6)	697 641.8 (906 302.4)	560 252.8 (454 482.8)	681 188.8 (672 673.6)
*Nursing homes*								
For-profit nursing homes, mean % (SD)	71.2 (10.6)	71.7 (13.0)	61.5 (14.1)	60.1 (23.1)	77.3 (6.3)	64.5 (16.8)	62.3 (22.4)	66.1 (14.7)
Occupancy rate, mean % (SD)	81.9 (7.0)	79.0 (7.5)	83.0 (6.0)	85.0 (8.0)	79.6 (6.8)	75.7 (8.3)	80.3 (6.0)	82.0 (6.3)
Total number of beds, mean N (SD)	28 037.4 (22 476.5)	45 019.2 (39 163.1)	30 797.5 (35 297.5)	32 228.0 (30 144.7)	38 322.3 (25 276.8)	26 684.0 (38 181.5)	28 339.4 (25 211.5)	36 455.8 (32 645.9)
*Income*								
Median income, mean $ (SD)	46 886 (7532)	50 411 (8563)	51 625 (6431)	59 560 (7610)	56 741 (6340)	61 700 (9826)	68 322 (11 500)	72 737 (9991)
Percent below poverty level, mean % (SD)	17.6 (3.3)	15.8 (3.1)	14.3 (2.3)	13.0 (2.7)	14.7 (2.5)	12.4 (2.3)	11.1 (2.8)	10.8 (1.4)

**Figure 2.**
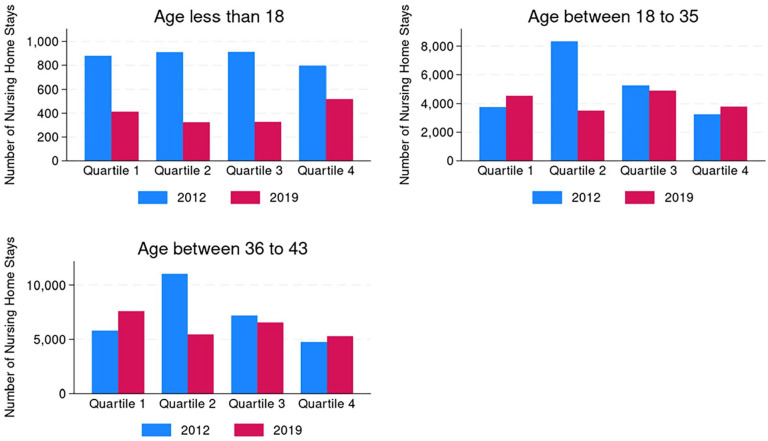
Total nursing home stays by age group by quartile of HCBS expenditures per state resident with disability, 2012 and 2019. *Note.* 2012 Quartile 1, $710 to $1069; 2012 Quartile 2, $1070 to $1687; 2012 Quartile 3, $1688 to $2338; 2012 Quartile 4, $2339 to $6109; 2019 Quartile 1, $784 to $1145; 2019 Quartile 2, $1146 to $1825; 2019 Quartile 3, $1826 to $2917; 2019 Quartile 4, $2918 to $6541.

In Figure 2, we present nursing home stays by age group for each quartile of HCBS spending. In 2012, the states in the third quartile of HCBS spending had the highest number of nursing home stays among children (912 stays), and the states in the second quartile of HCBS spending had the highest number of nursing home stays for adults (age 18-35: 8325 stays, age 36-43: 11 031 stays). In 2019, states in the top quartile had the highest number of nursing stays among children (519 stays). Among adults aged 18 to 35, states in the third quartile of spending had the highest number of total nursing home stays (4907 stays), while among adults aged 36 to 43, states in the bottom quartile had the highest number of nursing home stays (7607 stays).

**Figure 3.**
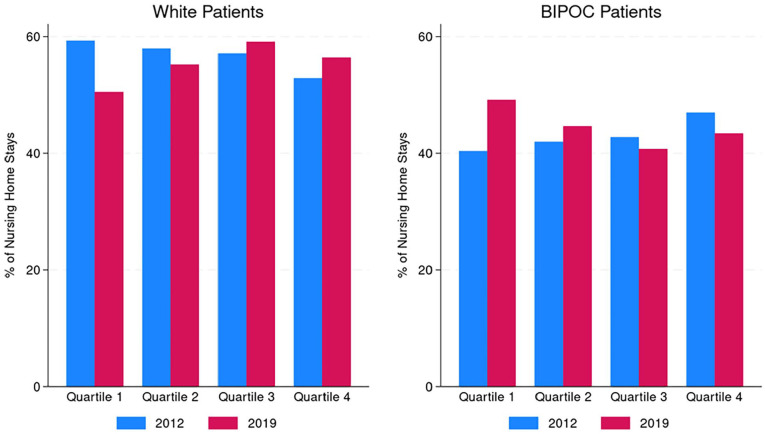
Proportion of nursing home stays by race by quartile of HCBS expenditures per state resident with disability, 2012 and 2019. *Note.* 2012 Quartile 1, $710 to $1069; 2012 Quartile 2, $1070 to $1687; 2012 Quartile 3, $1688 to $2338; 2012 Quartile 4, $2339 to $6109; 2019 Quartile 1, $784 to $1145; 2019 Quartile 2, $1146 to $1825; 2019 Quartile 3, $1826 to $2917; 2019 Quartile 4, $2918 to $6541.

We present the percentages of nursing home stays by race/ethnic group for each quartile of HCBS spending in Figure 3. The percentage of nursing home stays by BIPOC patients was similar across the quartiles, ranging from 40% to 47% in 2012 and from 41% to 49% in 2019.

### Regression Results

Among all individuals under the age of 44, higher HCBS spending was associated with fewer short-term stays and more long-term care stays, but the association was not statistically significant in the unadjusted and in the adjusted model (Table 2).

**Table 2.** Association Between a Change in HCBS Expenditures and Nursing Home Utilization.

Age < 44	N	Unadjusted coefficients (Model 1)		Adjusted coefficients (Model 2)	
		HCBS expenditure per individual with a disability [C.I. 95%]	*P*-value	HCBS expenditure per individual with a disability [C.I. 95%]	*P-*value
Short Stay					
Overall	229 484	0.1 [−19.5 to 19.7]	.994	−3.5 [−21.9 to 14.9]	.706
NHW	128 827	−8.6 [−19.6-−2.3]	.120	−9.9 [−21.0 to 1.2]	.079
BIPOC	100 215	8.6 [−2.3 to 19.5]	.118	6.2 [−3.7 to 16.2]	.213
Long stay					
Overall	169 207	6.4 [−20.8 to 33.6]	.638	6.0 [−17.6 to 29.5]	.614
NHW	91 585	−0.7 [−16.2 to 14.9]	.933	−1.0 [−14.3 to 12.2]	.874
BIPOC	77 476	7.0 [−6.1 to 20.1]	.289	7.0 [−5.5 to 19.4]	.267
Age < 18		HCBS expenditure per disability [C.I. 95%]	*P*-value	HCBS expenditure per disability [C.I. 95%]	*P-*value
Short stay					
Overall	8297	−6.7 [−12.1 to −1.3]	.016	−6.3 [−11.3 to −1.4]	.013
NHW	5364	−5.1 [−9.0 to −1.1]	.013	−5.0 [−8.8 to −1.3]	.010
BIPOC	2911	−1.6 [−3.9 to 0.7]	.159	−1.3 [−3.5 to 0.8]	.224
Long stay					
Overall	10 504	−4.1 [−7.3, 0.5]	.093	−3.4 [−7.3, 0.5]	.084
NHW	3859	−2.1 [−3.7 to −0.4]	.015	−1.9 [−3.1 to −0.2]	.012
BIPOC	6638	−2.0 [−5.6 to 1.6]	.268	−1.5 [−4.4 to 1.4]	.304
Age 18-43		HCBS expenditure per disability [C.I. 95%]	*P*-value	HCBS expenditure per disability [C.I. 95%]	*P*-value
Short stay					
Overall	221 187	6.8 [−12.7 to 26.3]	.489	2.9 [−14.9 to 20.6]	.746
NHW	123 463	−3.6 [−13.1 to −5.9]	.454	−4.9 [−14.8 to 5.0]	.324
BIPOC	97 304	10.2 [−1.8 to 22.3]	.095	7.6 [−3.0 to 18.1]	.156
Long stay					
Overall	158 703	10.5 [−16.2 to 37.1]	.433	9.4 [−13.4 to 32.1]	.412
NHW	87 726	1.4 [−13.8 to 16.6]	.854	0.8 [−12.0 to 13.6]	.895
BIPOC	70 838	9.0 [−4.2 to 22.2]	.178	8.5 [−3.7 to 20.7]	.169
Other variables*		No		Yes	
State and year FE		Yes		Yes	
Clustered SE		Yes		Yes	

*Note.* *Model 2 adjusts for the total number of residents with a disability, the total number of beds across all nursing facilities, percentage of for-profit nursing homes, nursing home occupancy rate, median income, and percent of population below the poverty line.

Among children, an increase in HCBS spending was associated with fewer nursing home stays. A $1000 increase in HCBS spending per resident with a disability was associated with a small but significant decrease in short-term nursing home stays (6.7 fewer stays; 95% CI: −12.1 to −1.3; *P* = .016). The effect was concentrated among children who were NHW (5.1 fewer stays; 95% CI: −9.0 to −1.1; *P* = .013). For long-term stays, higher HCBS spending was associated with lower nursing home use among NHW patients (2.1 fewer stays; 95% CI: −3.7 to −0.4; *P* = .015). However, among BIPOC patients, we did not find statistically significant association of nursing home use and HCBS spending (2.0 fewer stays; 95% CI: −5.6 to 1.6; *P* = .268). Among adults aged 18 to 43, we did not find a statistically significant association between HCBS spending and nursing home use for either NHW or BIPOC patients (Table 2).

The results were similar after adjusting for the total number of residents with a disability, the total number of beds across all nursing facilities, percentage of for-profit nursing homes, nursing home occupancy rate, median income, and percent of population below the poverty line (Table 2).

In our sensitivity analyses, where we excluded states with high levels of missing expenditure data, we find that higher HCBS spending was associated with fewer short-term stays among all individuals under the age of 44 (13.8 fewer stays; 95% CI: −26.1 to −1.5; *P* = .029; Appendix Table A1). Other results were consistent with the main findings. Specifically, a $1000 increase in HCBS spending per resident with a disability was associated with decrease in short-term nursing home stays (5.3 fewer stays; 95% CI: −9.1 to −1.5; *P* = .007) and long-term nursing home stays among NHW children (1.8 fewer stays; 95% CI: −3.3 to −0.4; *P* = .015; Appendix Table A1).

## Discussion

On average, states with higher HCBS expenditures per residents with a disability had fewer short-term and long-term nursing home stays among NHW children. These findings reflect not only considerable heterogeneity in the unmet needs, preferences for care, and resources available to individuals under the age of 44 with complex care needs, but also represent an example of how race-neutral policy decisions may contribute to worsening racial and ethnic disparities.

One possible explanation of the inverse relationship between HCBS spending and nursing home use among children but not among adults is that school-aged children with complex medical needs receive substantial additional support through federally mandated programs such as EPSDT or through school-based services (including physical and occupational therapy, instructional support and assistive technologies). The HCBS benefits may have an additive effect in reducing nursing home use in children. However, we observed this effect only for NHW children and not for BIPOC children. The observed disparities in nursing home use between BIPOC and NHW children may reflect disparities in access to skilled nursing and custodial care at home, regardless of eligibility and generosity of benefits.^8,28^

Moreover, we note that on average the total value of HCBS expenditures per individual with a disability for most states is low. In 2019, HCBS expenditures per resident with a disability ranged from $950 for the states in the bottom quartile of spending to $4394 for the states in the top quartile of spending. However, the observed changes in spending within states over this time period were small and the differences may not be sufficient to have an impact on nursing home use. In other words, the loss of federal and school-based benefits for children with complex medical needs as they reach early adulthood may lead to nursing home use even in states that increase HCBS spending.

Although HCBS aim to reduce long-term nursing home use and do not specifically target short-term skilled nursing and rehabilitative needs, we observed that higher HCBS spending was associated with fewer short-term nursing home stays among NHW children. There may be some spillover effects from HCBS to short-term post-acute care utilization. Our findings complement a recent study of individuals dually enrolled in Medicare and Medicaid that found an association between the use of HCBS prior to hospitalization and lower likelihood of short-stay nursing home use after hospital discharge as well as shorter length of nursing home stays among individuals discharged to post-acute care in a nursing home.^29^ Another possible explanation is that states with higher HCBS spending may also have higher spending on other services to meet patient needs for short-term post-acute care in the community. Future research should evaluate these potential mechanisms and their implications for racial/ethnic disparities in at home care for children and young adults with complex clinical needs. For instance, a 2023 systematic review of the literature on disparities in Medicaid HCBS noted a dearth of research on acceptability of HCBS among marginalized groups and underscored the need for research on the interplay between availability, access, accommodation, and acceptability of HCBS in diverse communities.^30^

### Limitations

Our study has several limitations. First, information on race was obtained from the MDS, a commonly used source that has not been externally validated. Second, some racial/ethnic groups were not well represented in the population. Since the sample sizes of the Black/African American and Hispanic group comprise a majority of the BIPOC group, our results may not be generalizable to all racial and ethnic minoritized subgroups. Third, in this observational study we were unable to account for patient and caregiver preferences, or other potential confounders of nursing home use among children and young adults. Fourth, the total number of residents with disabilities was collected form the American Community Survey which includes hearing and vision difficulty, in addition to cognitive, ambulatory, self-care, and independent living difficulty, in its definition of disability. Moreover, we were unable to disaggregate expenditures by age group. Hence, our estimates of HCBS spending per individual with disability may under- or overestimate actual expenditures for the target population. Fifth, our findings reflect healthcare use prior to the COVID-19 pandemic and may not be generalizable to more recent periods. Finally, our studied population reflects the changes in nursing home use among children and adults aged between 18 and 43 as opposed to those who need or are eligible for HCBS services. It is possible that, given improved medical technologies, more children with significant functional and cognitive needs are living into adulthood. This may mean that the prevalence of individuals who need HCBS and nursing home care is increasing among adults 18 to 43 years old.

## Conclusions

Our findings highlight a persistent need for alternatives to nursing home placement among children and adults with complex behavioral, cognitive, and functional needs. Investments in HCBS are necessary to reduce nursing home use among younger adults. However, to mitigate racial disparities in nursing home use among children, HCBS spending alone may not be sufficient. Interventions that target causes of racial disparities in caregiving for medically complex younger individuals such as access to advocacy groups, personal care services, and language barriers, are urgently needed to achieve equitable outcomes in this vulnerable patient population.

## Appendix

**Table A1.** Association Between a Change in HCBS Expenditures and Nursing Home Utilization (Excluding States With Missing Data).

Age < 44	N	Adjusted* Coefficients	
		HCBS Expenditure per Individual with a Disability [C.I. 95%]	*P*-value
Short Stay			
Overall	193 191	−10.4 [−28.6 to 7.8]	.258
NHW	111 994	−13.8 [−26.1 to −1.5]	.029*
BIPOC	80 840	3.2 [−6.2 to 12.6]	.493
Long stay			
Overall	142 853	−1.3 [−15.0 to 12.4]	.849
NHW	80 949	−5.9 [−13.8 to 1.9]	.136
BIPOC	61 776	4.6 [−4.8 to 14.0]	.329
Age < 18		HCBS expenditure per disability [C.I. 95%]	*P-*value
Short stay			
Overall	7426	−5.9 [−10.4 to −1.4]	.011*
NHW	4924	−5.3 [−9.1 to −1.5]	.007**
BIPOC	2480	−0.6 [−1.8 to 0.6]	.306
Long stay			
Overall	9071	−2.2 [−4.6, 0.2]	.075
NHW	3572	−1.8 [−3.3 to −0.4]	.015*
BIPOC	5492	−0.3 [−1.7 to 1.1]	.643
Age 18-43		HCBS expenditure per disability [C.I. 95%]	*P*-value
Short stay			
Overall	185 765	−4.4 [−20.5 to 11.6]	.581
NHW	107 070	−8.5 [−19.3 to 2.4]	.122
BIPOC	78 360	3.8 [−4.9 to 12.6]	.385
Long stay			
Overall	133 782	0.9 [−11.6 to 13.4]	.887
NHW	77 377	−4.1 [−11.3 to 3.1]	.257
BIPOC	56 284	4.9 [−4.0 to 13.8]	.270
State and year FE		Yes	
Clustered SE		Yes	

*Note.* We adjusted for the total number of residents with a disability, the total number of beds across all nursing facilities, percentage of for-profit nursing homes, nursing home occupancy rate, median income, and percent of population below the poverty line.
